# *ESR1* alterations and metastasis in estrogen receptor positive breast cancer

**DOI:** 10.20517/2394-4722.2019.12

**Published:** 2019-05-04

**Authors:** Jonathan T. Lei, Xuxu Gou, Sinem Seker, Matthew J. Ellis

**Affiliations:** 1 Interdepartmental Graduate Program in Translational Biology & Molecular Medicine, Baylor College of Medicine, Houston, TX 77030, USA.; 2 Lester and Sue Smith Breast Center, Baylor College of Medicine, Houston, TX 77030, USA.; 3 Departments of Medicine and Molecular and Cellular Biology, Baylor College of Medicine, Houston, TX 77030, USA.

**Keywords:** Endocrine therapy resistance, ESR1 fusions, ESR1 mutations, breast cancer, metastasis

## Abstract

Endocrine therapy is essential for the treatment of patients with estrogen receptor positive (ER+) breast cancer, however, resistance and the development of metastatic disease is common. Understanding how ER+ breast cancer metastasizes is critical since the major cause of death in breast cancer is metastasis to distant organs. Results from many studies suggest dysregulation of the estrogen receptor alpha gene (*ESR1* ) contributes to therapeutic resistance and metastatic biology. This review covers both pre-clinical and clinical evidence on the spectrum of *ESR1* alterations including amplification, point mutations, and genomic rearrangement events driving treatment resistance and metastatic potential of ER+ breast cancer. Importantly, we describe how these *ESR1* alterations may provide therapeutic opportunities to improve outcomes in patients with lethal, metastatic breast cancer.

## INTRODUCTION

Breast cancer is one of the leading cancer-related causes of death worldwide with more than one million new cases and more than 450,000 deaths per year according to the World Health Organization. About 70% of diagnosed cases express estrogen receptor alpha (ER)^[1]^ , where ER signaling is the defining and driving event contributing to tumor growth and disease progression in these ER+ breast tumors.

**Figure 1:**
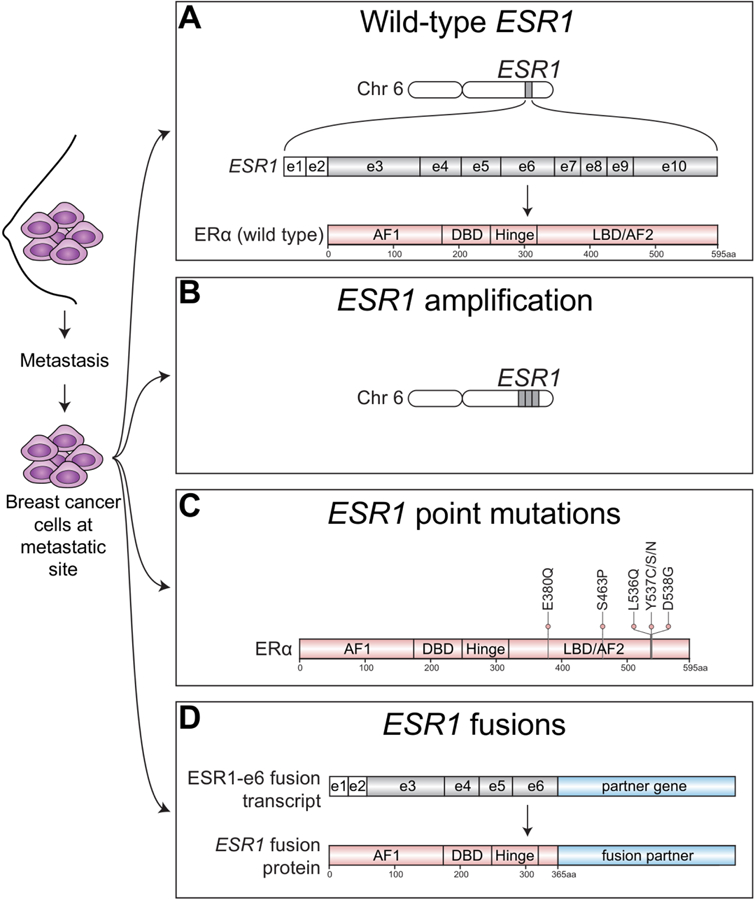
Spectrum of *ESR1* alterations found in metastatic ER+ breast cancer. ER+ breast cancer cells that have spread beyond the breast to metastatic sties have been found to express wild-type ESR1 or harbor a variety of *ESR1* alterations. A: Metastatic tumors can express wild-type estrogen receptor alpha protein (ERa), which is encoded by the estrogen receptor alpha gene (*ESR1* ) located on chromosome (chr) 6. *ESR1* transcripts are generated from 2 non-coding exons (e) depicted by white boxes and 8 coding exons depicted by gray shaded boxes; B: Metastatic ER+ tumors may also harbor amplification of *ESR1* resulting in multiple copies of *ESR1* and increased ER protein expression; C: Point mutations that cluster within the ligand-binding domain (LBD) of *ESR1* that confer constitutive ligand-independent activation of *ESR1* mutants have also been well-described in metastatic ER+ breast tumors, especially those which had been extensively pretreated with AIs; D: Emerging studies have now identified structural rearrangements involving *ESR1* that generate in-frame *ESR1* fusion transcripts. In-frame fusion transcripts that retain the first 6 exons of *ESR1* (*ESR1*-e6) produce stable *ESR1* fusion proteins have been shown to be transcriptionally active and drive endocrine therapy resistance and metastasis in ER+ breast cancer. AF1: activation function 1 domain; DBD: DNA-binding domain; AF2: activation function 2 domain; aa: amino acid

ER is a transcription factor consisting of various functional domains encoded by *ESR1* located on chromosome 6 [Figure 1A]. *ESR1* transcripts are generated by 2 non-coding and 8 exons that specifies protein-coding domains. The N-terminal activation function 1 (AF1) domain functions in a hormone-independent manner and is post-translationally modified by phosphorylation events that increase transcriptional and pathogenic activity^[2–5]^. The DNA-binding domain (DBD) contains two zinc finger motifs responsible for binding to estrogen response element (ERE) DNA sequences within the enhancers and promoters of ER target genes. The C-terminal domains include the ligand-binding domain (LBD) and ligand-dependent activation function 2 (AF2) domain required for dimerization and transactivation. The LBD is required not only for estrogenic ligands but is also the domain that controls responses to anti-estrogen antagonists. The hinge domain contains the nuclear localization sequence and connects the activity from the ligand-independent AF1 and ligand-dependent AF2 together to fully promote activation of ER^[6]^.

Standard-of-care endocrine therapies that target ER itself include selective estrogen receptor modulators (SERMs), such as tamoxifen, and selective estrogen receptor degraders (SERDs), such as fulvestrant, that bind to the LBD. Aromatase inhibitors (AIs), such as letrozole, anastrozole, and exemestane, block the production of estrogens from androgens resulting in lower levels of circulating estrogen in the body. Despite the success of these agents in reducing relapse rates when given prophylactically after breast surgery and chemotherapy (adjuvant treatment), endocrine therapy resistance and the development of lethal metastatic disease is common and a major clinical problem. A major clinical feature of the disease is the long-term persistence of disseminated tumor cells despite endocrine therapy, with relapse risk continuing for decades after diagnosis^[7]^. The etiology of endocrine therapy resistance is complex and tremendous efforts have been made to uncover diverse mechanisms^[8]^.

Downstream signaling events from aberrantly activated growth factor receptor tyrosine kinases (RTKs) such as epidermal growth factor receptor (EGFR) and HER2 (*ERBB2*) have been shown to phosphorylate and increase ER transcriptional activity in a hormone-independent manner^[9]^. ER+ tumors that exhibit *ERBB2* amplification have reduced ER expression, reduced sensitivity to ER targeted therapies, and poor outcomes^[10]^. Nonetheless, co-targeting ER+/HER2+ breast cancer has been clinically successful. Experimental models have extended these ideas to other RTKs that are expressed by ER+ breast cancer. Interestingly, these investigations revealed a non-genomic or transcription-independent function of ER in association with EGFR^[11]^ and insulin-like growth factor receptor (IGF1-R)^[12]^ at the plasma membrane. However, clinical trials testing the use of EGFR inhibitors in endocrine treatment resistant ER+ breast cancer have produced modest or negative results^[13]^ suggesting that further insight into underlying mechanisms for RTKs and ER interactions are required for successful translation of this aspect of ER function.

Since *PIK3CA* is the most frequently mutated gene in ER+ breast cancer^[14]^, targeting components of the PI3K-AKT-mTOR pathway has also been described to treat endocrine refractory disease. Preclinical models demonstrated enhanced activation of the PI3K pathway in long-term estrogen deprived (LTED) ER+ breast cancer cells and a negative feedback system by which PI3K inhibition increases ER activity, potentially explaining the effectiveness of combinatorial mTOR and ER inhibition^[15]^. The use of an mTOR inhibitor, everolimus, in combination with endocrine therapy, significantly improves progression-free survival (PFS) for patients failing previous endocrine therapies^[16]^, although side effects are severe and stratification of patients for this treatment is essential. Treatment with a pan-PI3K inhibitor, buparlisib, in combination with fulvestrant increased PFS with compared to fulvestrant alone in patients with ER+ locally advanced or metastatic breast cancer (BELLE-3 clinical trial)^[17]^. Greater benefit was observed in patients treated with buparlisib harboring *PIK3CA* mutations. However, significant toxicities in buparlisib treated patients have stopped further clinical trials of the drug in this setting. In contrast to pan-PI3K inhibitors, alpelisib, an agent that specifically targets the alpha isoform of *PIK3CA*, has been shown to overcome the toxicities associated with pan-PI3K treatment. Apelisib greatly improved PFS in patients when given in combination with fulvestrant to patients with endocrine-refractory, advanced ER+ breast cancer harboring *PIK3CA* mutations^[18]^. There was no significant benefit to PFS in patients with non-*PIK3CA* mutant tumors suggesting that *PIK3CA* status is a potential biomarker to predict response to PI3K inhibition. Results from studies also further suggest that targeting specific mutant isoforms of PI3K reduces toxicities leading to increased tolerability and therefore can be given for a longer duration compared to other pan-PI3K agents such as buparlisib.

Dysregulation of cell cycle components is common in ER+ breast cancer, particularly the Cyclin D-CDK4/6-Rb axis in the luminal B subtype^[19]^. This includes amplification of Cyclin D1 (*CCND1*), gene copy gain of *CDK4* and loss of negative regulators such as p16 and p18 (*CDKN2A* and *CDKN2C*)^[19]^. Together with downstream activity from tyrosine kinase growth factor signaling described earlier, these events promote phosphorylation of Rb and resistance to endocrine therapy^[20]^. CDK4/6 inhibitors such as palbociclib and ribociclib, are now FDA approved for use in combination with endocrine therapy to treat advanced stage ER+ disease. Other studies are now examining the use of such inhibitors to treat early stage ER+ disease in both neoadjuvant and adjuvant settings (ClinicalTrials.gov identifiers for PALLET NCT02296801 and PALLAS NCT02513394, respectively). Some trials have already reported promising results in the neoadjuvant setting^[21]^.

In addition to metastatic breast tumors expressing wild-type ER [Figure 1A], alterations in *ESR1* itself, such as *ESR1* amplifications have been identified in metastatic ER+ disease^[22]^ [Figure 1B] . Other *ESR1* alterations found in endocrine therapy resistant breast tumors include point mutations in the ligand-binding domain (LBD)^[23]^ [Figure 1C] that confer constitutive hormone-independent activation of ER and are now a well-described mutational mechanism identified in up to 40% of metastatic breast cancer cases^[24]^. These are especially enriched in tumors pretreated with aromatase inhibitors^[25]^. Emerging evidence now suggests that chromosomal rearrangement events involving *ESR1* are yet another *ESR1* mutational mechanism driving endocrine therapy resistance and metastatic disease progression [Figure 1D]. Hereon, we focus on the spectrum of *ESR1* aberrations underlying treatment resistance and metastasis in ER+ breast cancer.

## *ESR1* AMPLIFICATION

The copy number increase of a confined area of a chromosome is defined as gene amplification/gain [Figure 1B] which may result in protein overexpression of the amplified gene therefore driving tumor biology. For example, *ERBB2* amplification^[26]^ and fibroblast growth factor receptor 1 gene (*FGFR1*) amplification^[27]^ are drivers of therapeutic resistance and poor prognosis in ER+ breast cancer. The discovery of *ESR1* gene amplifications in 1990^[28]^ sparked intense interest in investigating the role of this mutational event to be a potential driver of endocrine therapy resistance and recurrent disease in ER+ breast tumors.

### Incidence of *ESR1* amplifications in ER+ breast cancer

*ESR1* amplification is found in up to 30% of ER+ breast tumors^[22,28–37]^ depending on the detection method and scoring systems^[38]^. A study by Holst *et al*.^[29]^ that analyzed over 2,000 breast tumors, showed that 20.6% of tumors harbored *ESR1* amplifications and 14% showed *ESR1* copy number gain by using fluorescence in situ hybridization (FISH) method and validated by quantitative PCR^[29]^. Nearly all *ESR1* amplified tumors in these samples also expressed high levels of ER protein by immunohistochemistry. Additional analysis from precancerous ductal and lobular carcinoma in situ (DCIS and LCIS) breast tumors showed over one-third of these samples also harbored *ESR1* amplifications suggesting that *ESR1* amplifications present in early-stage breast cancer may drive disease progression. Two other independent studies that also used FISH, both showed that *ESR1* amplification frequency is between 20%−22%^[34–35]^, consistent with Holst *et al*.^[29]^. In contrast, other studies by Brown *et al*.^[30]^, Horlings *et al*.^[31]^, Reis-Filho *et al*.^[32]^, and Vincent-Salomon *et al*.^[33]^, have shown a much lower frequency of *ESR1* amplifications, in which *ESR1* amplification or gain was less than 5% by using array comparative genomic hybridization (aCGH) and validated by FISH by the majority of these studies. Another study which used a multiplex ligation-dependent probe amplification (MLPA) approach to analyze 104 invasive breast cancers identified 16% of samples harbored *ESR1* amplifications consisting of low level gains^[36]^. A variation in the frequency of *ESR1* amplification found among metastatic breast samples has also been reported. A seminal study from Jeselsohn *et al*.^[37]^ examined *ESR1* amplification in the metastatic setting using next generation sequencing approaches. They reported the frequency of *ESR1* amplification in ER+ tumors at less than 2% in both the primary and metastatic setting^[37]^. Using NanoString sequencing approaches, a recent study reported that 13% of ER+ metastatic breast tumors harbored *ESR1* amplifications. Interestingly, the authors found an enrichment of *ESR1* amplifications in bone metastatic samples, suggesting that *ESR1* amplification may underlie organ-specific metastatic behavior of ER+ breast cancers^[39]^.

### Correlation between *ESR1* amplification, protein expression, and clinical significance

Many studies show positive correlation between *ESR1* amplification and ER protein expression suggesting that amplification may lead to production of elevated levels of oncogenic ER protein^[28,29,34,35]^. Interestingly, studies have shown that *ESR1* amplification in a subset of ER+ breast cancers were associated with tamoxifen resistance and poor prognosis^[40,41]^. In contrast, contradicting studies have identified *ESR1* amplification as an indicator of longer disease-free survival and increased sensitivity to tamoxifen treatment^[35,42]^. These conflicting results suggest that more dedicated studies will be required to fully understand the clinical implications of *ESR1* amplifications. Results from other studies have identified *ESR1* amplification in benign and early-stage breast cancer and is associated with endocrine therapy resistance. Discovery of *ESR1* amplifications in benign papillomas and early-stage breast cancer such as ductal hyperplasias suggests that *ESR1* amplifications may play a role in the tumor initiation process since high expression of ER in benign breast cells is associated with higher breast cancer risk^[29,43,44]^, but these findings still require further validation. The insignificant difference of *ESR1* amplification between invasive and non-invasive breast cancers suggests that *ESR1* copy number alteration might not be used as a key predictive marker for invasion and metastasis, however its enrichment in recurrent disease, especially after endocrine therapy treatment, suggests that it likely plays a role in intrinsic and/or acquired resistance to endocrine therapy and metastatic disease progression^[45–48]^.

Although the use of endocrine agents that block estrogen production (AIs) or block ER function (SERM/SERD) are front-line therapies to treat metastatic ER+ breast cancer, the use of high-dose estrogens has also been reported to be effective. This approach was first described over 70 years ago before the discovery of anti-estrogens to treat advanced breast cancer^[49]^ . More recently, a study reported a breast cancer patient harboring an *ESR1* amplification showed tumor regression in a liver metastasis after receiving estradiol treatment as a primary therapy ^[50]^. Another study using a patient-derived xenograft (PDX) model harboring an *ESR1* amplification derived from a patient with endocrine-refractory disease demonstrated that tumor growth was suppressed with estradiol treatment^[47]^. These results were corroborated in an independent study using a LTED ER+ MCF7 breast cancer cell model system in which such cells acquire *ESR1* amplification during long term estrogen deprivation showed an apoptotic response upon estradiol treatment^[48]^. Collectively, these studies suggest a role for *ESR1* amplification in driving endocrine therapy resistance and metastasis and that treating *ESR1* amplified tumors with intermediate doses of estradiol (6 mg daily) is an option for some patients.

The presence of *ESR1* amplification in some breast cancers is undeniable. However, a clear link between the presence of *ESR1* amplifications in breast tumors and endocrine therapy resistance and metastasis remains to be shown. Deeper multi-dimensional characterization of relapsed and/or metastatic breast tumors at the RNA, DNA, and protein levels may aid to better understand its prognostic value. Therefore, more studies will be required to better understand the functional and therapeutic significance of *ESR1* amplifications in driving endocrine therapy resistance and metastasis.

### *CYP19A1* amplification

While *ESR1* amplification has been an intense area of investigation underlying endocrine therapy resistance as described above, a study focusing on genomic aberrations of the drug target of AIs, aromatase (*CYP19A1*), has deepened our understanding of endocrine-refractory ER+ breast tumors. Copy number alterations in the gene encoding aromatase, *CYP19A1*, also has been shown to promote resistance to AIs in patients with metastatic ER+ breast cancer. While *CYP19A1* amplification is very rare in primary untreated ER+ breast cancers, Magnani *et al*.^[51]^ found that 21.5% of AI-refractory relapsed tumors to harbor *CYP19A1* amplification, suggesting that *CYP19A1* amplification is an acquired endocrine therapy resistance mechanism^[51]^. This study also revealed that both *CYP19A1* and *ESR1* were frequently co-amplified in AI treated patients, further suggesting that these two amplification events may function collaboratively. To better understand the role of *CYP19A1* amplification and endocrine therapy resistance, a LTED MCF7 ER+ breast cancer cell model was used which was found to acquire copy number alterations around the *CYP19A1* locus compared to parental cells MCF7 cells^[51]^ . Elevated levels of both *CYP19A1* mRNA and CYP19A1 protein were observed in *CYP19A1* amplified LTED cells compared to parental cells. The functional consequences of *CYP19A1* amplification in the LTED cells were increased aromatase activity, enhanced ER recruitment to regulatory regions on DNA of target genes and their transcriptional activation leading to reduced sensitivity to AI treatment ^[51]^. These results suggest that *CYP19A1* amplification, in addition to *ESR1* amplification, could potentially represent biomarkers of endocrine therapy resistance. More studies are needed to validate these findings in more patient datasets. Furthermore, deeper studies focusing on how these amplification events contribute to the metastatic behavior of endocrine-refractory ER+ breast tumors are needed. These results highlight the possibility that response to standard-of-care endocrine therapies are not only as a consequence of *ESR1* amplification but may also be critically dependent on the status of the target genes of endocrine therapies themselves.

## *ESR1* POINT MUTATIONS

When patients with ER+ breast cancer relapse, up to 15% have lost ER expression and therefore targeting ER in this population is likely to be ineffective, although false negative ER results are a concern if the ER analysis was conducted on bone biopsies exposed to acid formalin, or if the analysis was conducted on samples prone to degradation such as cells detected in pleural fluid. The remaining 85% of patients may initially benefit from first-line endocrine therapy, but metastatic disease progression due to acquired resistance is inevitable. One well-established mechanism explaining this relentless pattern of acquired endocrine therapy resistance is the acquisition of activating point mutations that cluster within the ligand-binding domain (LBD) of *ESR1* [Figure 1C]. Substitution of tyrosine at position 537 to serine (Y537S) in the LBD of *ESR1* was first reported to confer constitutive, ligand-independent activity of ER in experimental breast cancer models^[52]^. However, such mutations were not known to occur in human tumors until Fuqua *et al*.^[23]^ reported that estrogen-independent activation could be driven by another Y537 substitution, Y537N, that was identified in a metastatic sample from a breast cancer patient who experienced disease progression on hormonal therapy. This study also showed that Y537N was able to drive resistance to tamoxifen in experimental models.

### Frequent *ESR1* point mutations in endocrine-refractory, metastatic ER+ breast cancer

Advances in sequencing technologies have allowed more sensitive detection and thus insights into the landscape of *ESR1* LBD point mutations in both primary and metastatic ER+ breast tumors. Three *ESR1* mutations, Y537S, Y537N, and D538G were identified by next-generation sequencing in 14 out of 80 patient samples with endocrine-refractory, metastatic ER+ breast cancer^[53]^. Notably, all breast tumors from patients that were found to harbor *ESR1* LBD point mutations were treated with AIs. Interestingly, these alterations were not detected in matched primary samples and were also not detected in separate large sets of treatment naïve patients. Analysis of an independent ER negative (ER-) cohort also failed to detect any *ESR1* point mutations in the LBD^[53]^ . Although *ESR1* mutations were found in 3% of primary samples in this population, alterations in Y537 and D538 residues of *ESR1* were enriched in patients treated extensively with AIs^[53]^. These results suggest that these *ESR1* LBD mutations are acquired, or detected, in patients after treatment with endocrine therapy.

In addition to Y537 alterations, frequent amino acid substitution of aspartate 538 to glutamate (D538G) was identified in liver metastases from 5 out of 13 metastatic ER+ breast samples^[54]^. Another study which enrolled 11 metastatic ER+ breast cancer patients with exposure to serial endocrine therapies, identified that over half of these patient’s metastatic samples harbored *ESR1* mutations localized in the LBD, that included Y537S, Y537C, Y537N, D538G, and L536Q mutations^[55]^. Further evidence for the recurrent presence of Y537 and D538 mutations in the LBD of *ESR1* was shown in 9 out of 76 metastatic samples from patients with ER+ disease^[37]^. One patient from this study acquired a tyrosine substitution to cysteine mutation (Y537C) at the metastatic site, which was not detected prior to treatment^[37]^. Taken together, these studies indicate the most frequent *ESR1* LBD point mutations are those affecting Y537 and D538 residues. Furthermore, the presence of *ESR1* point mutations predominately appear in late-stage breast cancer patients that have been treated with multiple lines of endocrine therapies but rarely in treatment naïve cases. This strongly suggests a role for *ESR1* point mutations in acquired endocrine resistance and metastasis.

Although formalin-fixed paraffin-embedded tumor specimens are widely used for next generation sequencing to capture *ESR1* mutations used by studies as described above^[37,53,54]^, collection of plasma circulating DNA to detect *ESR1* mutations by droplet digital PCR (ddPCR) have now been implemented in several clinical trials^[56–59]^. Such “liquid biopsies” have shown that collecting circulating DNA samples maintains the genomic landscape of the primary tumor suggesting that less invasive detection methods may efficiently identify *ESR1* point mutations once the disease has become resistant to treatment and/or has become metastatic. Interestingly, Y537 and D538 substitutions were identified in 7% of ER+ primary tumors using ddPCR, which may lead us to review the conclusion that *ESR1* point mutations rarely exist in primary tumor, towards the idea that rare *ESR1* mutant sub-clones exist in primary breast tumors that become selected for over time^[60]^.

### Experimental models of *ESR1* point mutations

Several preclinical breast cancer models harboring *ESR1* LBD point mutations have been generated, providing research platforms to characterize the functional, transcriptional, and pharmacological properties of these mutations. ER point mutant proteins have been overexpressed by transfecting^[37,53,54]^ or transducing lentiviral vectors^[55,61]^ encoding *ESR1* mutant constructs into various ER+ breast cancer cell line models. The growth promoting properties of *ESR1* mutant expressing cell line models have shown that *ESR1* LBD mutants drive hormone-independent proliferation that is resistant to tamoxifen treatment^[23,37,47,53,54]^. Although fulvestrant efficiently inhibited the growth of point mutation bearing cells in a dose-dependent manner, growth was not reversed to levels of wild-type *ESR1* expressing cells^[37,47]^.

Since the expression of exogenous *ESR1* variant transcripts encoded by expression vectors is often initiated from non-endogenous human promoters that drive very high expression of constructs, it is unlikely to mimic the expression levels in human breast tumors harboring *ESR1* point mutations. To more accurately recapitulate tumor-related *ESR1* mutational events, CRISPR/Cas9 approaches have been utilized to knock in *ESR1* mutated sequences into ER+ breast cancer cells^[62,63]^. Both heterozygous and homozygous knock-in models have been shown to mediate resistance to endocrine therapies^[62,63]^.

Transcriptional properties of *ESR1* mutations in the LBD include their ability to drive constitutive hormone-independent transcriptional activation and enhance cell proliferation^[23,37,47,53–55]^. Human embryonic kidney 293T cells transfected with Y537C, Y537N, and D538G mutant constructs strongly activate an ERE-luciferase reporter in a ligand-independent manner compared to wild-type ER. Luciferase activity was unaffected by clinically relevant doses of tamoxifen and fulvestrant, however, high doses of these agents blocked *ESR1* mutant driven ERE-luciferase reporter activity^[37,53–55]^. These *ESR1* point mutations have also been shown to drive estrogen-independent activation of ER target genes in ER+ breast cancer cells^[37,53,54]^. The recruitment of ESR1-Y537S mutant to ER target genes and their expression driven by the mutant were further validated by ChIP-seq and RNA-seq^[62]^.

*ESR1* mutant-driven estrogen-independent tumor growth was also validated in both ER+ cell xenografts and patient-derived xenograft (PDX) models^[47,53]^. A PDX harboring ESR1-Y537S, WHIM20, has been generated from a patient with endocrine-refractory metastatic ER+ breast cancer that retains genomic features of the human counterpart^[47]^. This WHIM20 PDX model demonstrated estrogen-independent tumor growth^[47]^.

Despite such in-depth studies of transcriptional and growth-promoting properties endowed by *ESR1* LBD point mutations, the role of such mutations in driving cell invasion and tumor metastasis is underexplored. A scratch wound assay was performed on Y537S and D538G mutant expressing MCF7 cells to examine cell motility which showed enhanced cell migration under hormone-deprived conditions driven by these *ESR1* mutants^[54,61]^. A recent study sheds light on ER mutant-driven metastatic biology, showing a remarkable enrichment of metastasis-associated gene sets in *ESR1* mutant cells^[64]^. Consequently, Y537S and D538G mutant expressing MCF7 cells developed metastases after survival surgery to remove primary tumors in xenograft models. The Y537S mutant greatly potentiated both tumor growth and metastasis compared to D538G mutant^[64]^.

### Mechanisms and therapeutic vulnerabilities of breast cancers harboring *ESR1* point mutations

Structural analysis has revealed that the formation of hydrogen bonds between S537 or G538 and D351 located within helix 12 of *ESR1* LBD confers an agonist conformation to *ESR1* mutant proteins^[53]^. In wild-type ER, the binding of ligand alters the position of helix 12 into an open pocket, favoring recruitment of transcriptional coactivators such as p160 family members that include SRC-3, and histone acetylases CBP and p300. In contrast, tamoxifen results in disposition of helix 12 that hinders coactivators binding and results in recruitment of corepressors such as N-CoR/SMRT^[65]^. The substitution of D538 to glycine mimics the active conformation of wild-type ER bound by estrogen^[54]^.

To better understand the consequences of coactivator recruitment to mutant ER proteins, a proteomic profiling approach was used and revealed enhanced recruitment of transcriptional coactivators, histone H3 lysine 4 (H3K4) methyltransferase KMT2D/2C complex, as well as steroid receptor coactivators (SRCs), to ERE-bound ESR1-Y537S and ESR1-D538G mutants compared to ERE-bound wild-type ER^[66]^. Genetic inhibition of SRC-3 in HeLa cells expressing ESR1-Y537S and ESR1-D538G significantly suppressed activity of an ERE-luciferase reporter. Pharmacological inhibition using a pan-SRC inhibitor, SI-1, also suppressed transcriptional activation in *ESR1* mutant expressing HeLa cell lines and blocked cell proliferation in ER+ breast cancer cells stably expressing ESR1-Y537S and ESR1-D538G. Using a PDX naturally harboring the ESR1-Y537S mutation (WHIM20), treatment with an improved pan-SRC inhibitor, SI-2, suppressed growth *in vivo*. Suppression of WHIM20 tumor growth was even greater when SI-2 was administered in combination with an oral SERD, AZD9496, compared to either single agent alone, suggesting that targeting coactivator recruitment in combination with endocrine therapy could be a promising therapeutic strategy for breast tumors harboring *ESR1* LBD mutants such as Y537S and D538G^[66]^. Another study identified that the transcription factor TFIIH was also recruited by the ESR1-Y537S mutant^[62]^. Phosphorylation of Ser118 was found to be mediated by TFIIH kinase, cyclin-dependent kinase (CDK) 7 and subsequent ESR1-Y537S driven cell proliferation was suppressed by a CDK7 inhibitor, THZ1^[62]^. These results suggest that CDK7 may represent another target that is associated with ESR1 mutant proteins for therapeutic intervention.

Targeting non-genomic signaling pathways activated by *ESR1* mutants has also been investigated. As discussed above, interactions between ER with RTKs such as EGFR, HER2, and IGF1-R can activate downstream kinases. This results in phosphorylation of multiple transcriptional factors, including ER, and coregulators leading to changes in gene expression in a hormone-independent manner^[67]^. A recent study demonstrated that IGF1 signaling was the most activated pathway in *ESR1* mutant MCF7 cells^[61]^. IGF1 stimulation lead to increased phosphorylation of both IGF1-Rβ and insulin receptor substrate-1 (pIRS-1). Treatment with an IGF1-Rβ inhibitor (GSK1838705A) monotherapy was able to block Y537S-driven cell motility and combinatorial treatment with tamoxifen abrogated transcriptional activity and cell growth driven by Y537S, Y537N, and D538G mutants^[61]^ . These results suggest that targeting non-genomic signaling pathways activated by *ESR1* mutants may be an additional therapeutic strategy to block *ESR1* mutant driven breast tumors.

Fulvestrant is used to treat metastatic ER+ breast cancer patients who have developed resistance to AI and tamoxifen. In preclinical models, transcriptional activity and cell proliferation of *ESR1* LBD mutant cells are partially sensitive to fulvestrant, requiring higher doses of fulvestrant compared to controls^[37,47,63]^. Moreover, fulvestrant did not completely block transcriptional activity nor cell proliferation compared to control cells expressing wild-type *ESR1*. Of note, *ESR1* mutants showed differential responses to fulvestrant. Y537S required the highest dose to completely block transcriptional activity and cell proliferation compared to other mutants, D538G, E380Q and S463P^[63]^. Using an MCF7 xenograft model, *ESR1* mutants also showed differential responses to fulvestrant. Tumor growth of E380Q, S463P and D538G expressing tumors were significantly reduced while Y537S tumors showed resistance to treatment^[63]^. Given the inconvenience and poor bioavailability of intramuscular fulvestrant injections, second-generation SERDs, such AZD9496, that can be orally administrated have been tested and showed anti-proliferative ability in endocrine resistant experimental models cell xenograft models^[63,68]^. AZD9496 which has improved bioavailability compared to fulvestrant, was able to provide greater suppression of tumor growth in the Y537S MCF7 xenograft model and in a D538G PDX model compared to fulvestrant treatment^[63]^. A phase I clinical trial with AZD9496 in extensively pretreated advanced ER+ breast cancer patients has recently been completed with promising results, providing disease stabilization to the study cohort^[69]^. These results suggest that newer generation SERDs with improved bioavailability could be an attractive therapeutic option to treat endocrine-refractory breast tumors driven by *ESR1* mutations.

Treatment of late-stage ER+ breast cancer patients with CDK4/6 inhibitors in combination with endocrine therapy has been tremendously successful. CDK4/6 inhibitors have also been tested in PDX breast cancer models harboring *ESR1* point mutations. Wardell *et al.*^[70]^ reported the suppressive effects of a CDK4/6 inhibitor, palbociclib, on endocrine-refractory PDX tumors as long as the downstream target retinoblastoma (Rb) protein was expressed. Used as monotherapy or in combination with a hybrid SERM/SERD, bazedoxifene, palbociclib suppressed tumor growth of a WHIM20 PDX tumor harboring an ESR1-Y537S mutant. In contrast, palbociclib was ineffective in inhibiting the growth of WHIM43, a PDX naturally bearing ESR1-D538G mutant due to the lack of Rb protein expression, suggesting that Rb is a determinant of CDK4/6 treatment response. CDK4/6 inhibitors also showed favorable therapeutic effects in treatment-resistant ER+ patients harboring *ESR1* point mutations^[59]^.

Currently, screening of *ESR1* point mutations have not been used as biomarkers to predict response to therapy in the clinic. Wild-type ER, human epidermal growth factor receptor 2 (HER2), and progesterone receptor (PR), are histopathological markers that guide therapeutic selection. In clinical management of metastatic ER+ breast cancer, SERDs, such as fulvestrant is used for patients with resistance to AIs and tamoxifen without regard for *ESR1* mutation status. An analysis of BOLERO-2, a phase III clinical trial that enrolled ER+ breast cancer patients with locally advanced or metastatic disease whom progressed on AI, evaluated the prevalence of the two most frequent *ESR1* point mutations, Y537S and D538G and their effects on patient outcomes in ER+ metastatic patients^[56]^. Having either one or two of these mutations was associated with decreased overall survival. In the PALOMA-3 clinical trial which enrolled ER+ breast cancer patients with advanced, endocrine refractory disease, palbociclib combined with fulvestrant led to longer PFS than fulvestrant alone^[59,71]^. 69% of patients from the PALOMA-3 were analyzed for *ESR1* mutation status, which showed that 25% of these cases harbored *ESR1* mutations consisting mainly of Y537S, Y537N, D538G, and E380Q mutations^[59]^. However, palbociclib was found to provide equal benefit regardless of *ESR1* mutation status. Although these studies indicate that the presence of *ESR1* mutations may predict poor outcomes, they also highlight the need for more analyses of studies investigating the predictive value of *ESR1* mutation status and response to therapy once the disease has become endocrine therapy resistant.

The development of sequencing technologies and the various models to recapitulate *ESR1* mutant bearing tumors allow insightful studies into the landscape and targeted therapies of activating point mutations in the *ESR1* LBD. Further studies are needed to address the use of *ESR1* mutations as predictive biomarkers to stratify patient subsets and predict *ESR1* mutation specific therapeutic vulnerabilities.

### *ESR1* structural rearrangements and *ESR1* fusions

In contrast to well-studied *ESR1* point mutations, structural rearrangements involving *ESR1* are under-studied. A variety of *ESR1* gene fusion transcripts have been identified in luminal breast tumors^[72,73]^. Analysis of RNA-seq data from 990 primary TCGA breast samples revealed that 21 of these tumors (2.1%), all of the luminal B subtype, contained recurrent fusion transcripts involving the first two non-coding exons of *ESR1* fused to various C-termini sequences from the coiled-coil domain containing 170 gene, *CCDC170* (ESR1-e2>CCDC170)^[73]^. These fusion transcripts do not provide sufficient coding sequences to generate chimeric ER fusion proteins but instead generate truncated forms of CCDC170 proteins (∆CCDC170). Exogenous expression of ∆DCCDC170 in ER+ breast cancer cells led to enhanced growth and reduced sensitivity to tamoxifen^[73]^ suggesting a role for ESR1-e2>CCDC170 in endocrine therapy resistance. Another independent study that examined early stage and non-metastatic ER+ breast samples also identified two ESR1-e2>CCDC170 fusion transcripts as well as ESR1-e2>C6orf211 and another fusion containing the first 6 exons of *ESR1* fused to *AKAP12* (ESR1-e6>AKAP12)^[72]^. These *ESR1* fusions were identified in 4 out of 62 surgical samples (6.5%) that were resistant to letrozole aromatase inhibitor treatment 10–21 days post treatment as defined by Ki67 labeling^[74]^, suggesting a higher frequency for these *ESR1* fusions gene events in endocrine-refractory tumors compared to primary, untreated samples. However, detailed functional characterization and evidence demonstrating a causal role for *ESR1* fusions in endocrine therapy resistance has been lacking and the incidence of *ESR1* fusions from late-stage ER+ breast cancer still remains unclear. Furthermore, therapeutic strategies to treat *ESR1* translocated tumors remains poorly understood.

Using a PDX model to better understand endocrine therapy resistance, we previously reported a somatic gain-of-function event in the form of a chromosomal translocation identified in a patient presenting with aggressive endocrine therapy resistant, metastatic ER+ disease. This translocation produced an in-frame fusion gene consisting of exons 1–6 of *ESR1* (ESR1-e6) and the C-terminus of the Hippo pathway coactivator gene, *YAP1* (ESR1-e6>YAP1), thereby generating a stable *ESR1* fusion protein that was a highly active constitutive transcription factor^[47]^ [Figure 1D]. Our group more recently discovered another in-frame *ESR1* fusion gene involving the protocadherin 11 X-linked gene, *PCDH11X* (ESR1-e6>PCDH11X) provided by inter-chromosomal translocation that also produced stable *ESR1* fusion protein identified in a patient with endocrine-refractory, metastatic ER+ breast cancer^[75]^. In both ESR1-e6>YAP1 and ESR1-e6>PCDH11X fusions, the LBD of *ESR1* is replaced with in-frame sequences from another gene, and therefore the drug binding domain that endocrine therapies recognize is absent. These two fusions promoted endocrine therapy resistant cell proliferation and constitutively activated ER target genes. Interestingly, both fusions also upregulated an epithelial-to-mesenchymal transition (EMT)-like transcriptional signature, induced cell motility, and increased lung metastatic frequency^[75]^. These results suggest that *ESR1* fusions are able to drive not only endocrine therapy resistance, but also drive metastasis, linking these two lethal processes together.

Importantly, *ESR1* fusion-driven growth could be suppressed by CDK4/6 inhibition. This suggests that targeting downstream kinases of ER could be a potential therapeutic strategy to treat *ESR1* translocated tumors and further suggests that *ESR1* fusion status may be a potential biomarker to stratify patients to CDK4/6 inhibitor therapy. To further explore therapeutic strategies to target *ESR1* fusions, a collaborative study was performed to examine interacting proteins with *ESR1* fusion transcriptional complexes^[66]^ . Results from that study showed enhanced recruitment of 26S proteasomal subunits to ESR1-e6>YAP1 driving transcriptional activation and cell proliferation. Subsequent pharmacological inhibition with a broad-spectrum proteasome inhibitor, MG132, blocked ESR1-e6>YAP1-mediated activation of an ERE-luciferase reporter. Furthermore, bortezomib, a specific 26S proteasome inhibitor in phase II clinical trial used to treat endocrine-refractory, metastatic ER+ breast cancer in combination with fulvestrant^[76]^ suppressed growth driven by ESR1-e6>YAP1. Taken together, these results suggest that downstream ER kinases such as CDK4/6 as well as transcriptional coregulators such as the 26S proteasome are attractive therapeutic targets to treat *ESR1* fusion positive, metastatic breast tumors.

Additional in-frame *ESR1* translocations with diverse partner genes have now been identified in late-stage, endocrine-refractory, ER+ metastatic cases. These include ESR1-e6>DAB2, ESR1-e6>GYG1, and ESR1-e6>SOX9^[77]^. Like the ESR1-e6>YAP1 and ESR1-e6>PCDH11X fusions, the ESR1-e6>DAB2 and ESR1-e6>GYG1 fusions produce stable *ESR1* fusion proteins and all three were able to drive hormone-independent activation of a ERE-luciferase reporter^[77]^. Remarkably, these *ESR1* fusions all follow a pattern preserving the first six exons of *ESR1*, containing the N-terminal DNA binding domain fused in-frame to C-terminal partner genes, thus excluding the LBD in *ESR1* [Figure 1D]. Therefore, these additional *ESR1* fusion proteins likely drive pan-endocrine therapy resistance like our previously discovered ESR1-e6>YAP1 and ESR1-e6>PCDH11X fusions^[75]^. The functional and therapeutic significance of these additional *ESR1* fusions are the focus of ongoing investigation by our group and others.

In contrast to transcriptionally active *ESR1* fusions, we also identified an in-frame ESR1-e6 fusion, ESR1-e6>NOP2 in a treatment naïve primary breast tumor that was transcriptionally inactive despite producing stable *ESR1* fusion protein^[75]^ . ESR1-e6>NOP2 did not promote endocrine therapy resistant growth and was found to bind relatively few sites in a genome-wide DNA binding assay, potentially explaining the weak functional activity measured by our experimental systems. In addition, out-of-frame *ESR1* fusions identified in primary tumors preserving diverse exons of *ESR1* gene, ESR1-e3, ESR1-e4, ESR1-e5, and ESR1-e6 did not facilitate estrogen-independent proliferation^[75]^. More studies are required to fully understand the contribution of transcriptionally inactive in-frame and out-of-frame *ESR1* fusions in breast cancer.

*ESR1* fusion structural studies revealed that driver *ESR1* fusions from metastatic patients follow the same fusion pattern containing the first 6 exons of *ESR1* (ESR1-e6) fused to C-termini of diverse gene partners suggesting this pattern is strongly connected to endocrine therapy resistant, metastatic ER+ breast tumors. The observation of a highly consistent and recurrent *ESR1* breakpoint, together with the promiscuity of *ESR1* for a variety of fusion partners is certainly interesting. In prostate cancer, recurrent fusions involving promoter regions of an androgen regulated gene, transmembrane protease serine 2 gene (*TMPRSS2*) fused to coding sequences of erythroblastosis virus E26 gene (*ETS*) family members have been identified in more than 50% of prostate cancer cases^[78]^. Androgen receptor (AR) signaling has been shown to bring the androgen regulated gene *TMPRSS2* and the *ERG* gene in close proximity in prostate cancer cell line models^[79]^ . Androgen signaling also generates DNA damage in the form of double strand breaks (DSBs) at sites of TMPRSS2-ERG genomic breakpoints. These DSBs have been shown to be mediated by the class II topoisomerase beta, TOP2B, which is recruited to AR, inducing DSBs^[80]^. TMPRSS2-ERG gene fusions can then arise from dysfunction of mechanisms to repair DSBs, such as homologous recombination (HR) pathway and the error-prone non-homologous end-joining (NHEJ) pathway. AR-mediated DSBs in prostate cancer may provide clues to the recurrent *ESR1* breakpoints for *ESR1* fusions seen in breast cancer. Recruitment of TOP2B to ER and subsequent DSBs have been shown to occur at regulatory regions of ER target genes as a consequence of ER-mediated transcriptional activation^[81]^. Since regulatory regions of *ESR1* itself has also been shown to be bound by ER^[82]^, transcription-induced DSBs by ER, coupled with dysregulation of DSB repair mechanisms may contribute to the highly recurrent *ESR1* breakpoints. Although none of the fusion partners from endocrine-refractory, metastatic disease observed in our studies are known ER targets, additional studies are needed to better understand the diversity of preferred *ESR1* partner genes.

*ESR1* fusions that contain the first six exons of *ESR1* fused in-frame to partner genes are almost exclusively observed in endocrine therapy resistant, metastatic ER+ breast cancer, with the exception of ESR1-e6>NOP2, as described above, likely suggesting a role in driving disease pathogenesis. However, very few functionally significant *ESR1* fusions have been studied to date and therefore *ESR1* fusion events remains an understudied form of somatic mutation in breast cancer. The incidence of *ESR1* fusions is also still not well understood, especially in the metastatic setting, but the studies discussed here collectively suggest *ESR1* fusions to be present in at least 1% of metastatic breast cancer cases^[77]^, with the actual frequency likely to be higher as more studies on *ESR1* fusions emerge. Additional studies on *ESR1* fusions will further support the causal role *ESR1* fusions and have significant diagnostic and clinical implications since pathogenic *ESR1* fusions could be used as biomarkers to stratify patients for individualized healthcare in ER+ breast cancer. Therapeutic vulnerabilities from *ESR1* translocated tumors could be an alternative to chemotherapy in patients with rapidly progressing, endocrine therapy resistant disease.

## CONCLUSION

Endocrine therapy resistance and metastasis in ER+ breast cancer patients remain significant clinical problems. This review has focused on studies describing a spectrum of *ESR1* alterations including amplification, point-mutations, and structural rearrangements in endocrine-refractory, metastatic ER+ breast cancer cases. Results from these studies have provided insights into the underlying mechanisms that contribute to endocrine therapy resistance and metastasis.

Amplification of the *ESR1* locus results in overexpression of oncogenic ER protein in the breast and potentially reducing sensitivity of *ESR1* amplified breast tumors to endocrine therapies and therefore likely leads to disease progression and metastasis. Point mutations in the LBD of *ESR1*, the most common of which are Y537S and D538G, confer an agonist confirmation to such *ESR1* mutant proteins resulting in constitutively active mutant ER transcription factors that lead to activation of ER target genes in a hormone-independent manner while also promoting activation of metastasis-associated genes^[64]^. The finding that ER LBD mutant proteins are constitutively active in an estrogen-independent manner suggest that therapeutic strategies which work by blocking estrogen production, such as ovarian ablation and treatment with AIs, are likely to be ineffective in breast tumors harboring *ESR1* point mutations. Indeed, a significant proportion of *ESR1* LBD point mutations were identified in metastatic tumors that were extensively treated with AIs, suggesting that such mutations may be enriched in breast tumors upon AI treatment^[53]^. ESR1-Y537S and ESR1-D538G are partially sensitive to fulvestrant^[37,47,63]^, and newer oral SERDs that have better bioavailability compared to fulvestrant, such as AZD9496, have shown promising results in treating tumor growth driven by *ESR1* LBD point mutants in experimental models^[66]^. Although fulvestrant is used exclusively in the metastatic setting for ER+ disease, treating primary breast tumors upfront with fulvestrant or more potent SERDs like AZD9496 may reduce the incidence of disease driven by *ESR1* LBD point mutations.

Despite the potential effectiveness of fulvestrant in targeting ER proteins with point mutations in the LBD, it is completely ineffective against ER fusion proteins generated from in-frame *ESR1* fusion transcripts arising from *ESR1* translocations^[75]^. These *ESR1* fusions transcripts, ESR1-e6>YAP1 and ESR1-e6>PCDH11X, were identified in patients with metastatic ER+ breast tumors that were pan-endocrine therapy resistant^[75]^. Both fusions retain the first 6 exons of *ESR1* fused in-frame to C-terminal sequences of the partner gene but lack exons encoding the LBD, rendering these fusions insensitive to all endocrine therapies that target the LBD, including fulvestrant and most likely AZD9496. These *ESR1* fusions were found to generate hyperactive *ESR1* fusion proteins that not only drive endocrine therapy resistant growth, but also play a role in the metastatic process, reprogramming the ER cistrome to drive EMT and metastasis to lung^[75]^. Despite the lack of an *ESR1* LBD, blocking signaling downstream of *ESR1* fusions with a CDK4/6 inhibitor, palbociclib, suppressed *ESR1* fusion-driven growth at primary and metastatic sites in experimental models^[75]^. Similar to *ESR1* point mutations, *ESR1* fusion formation is likely a mechanism of acquired endocrine therapy resistance. To date, *ESR1* fusion transcripts that produce stable *ESR1* fusion proteins have only been detected in metastatic breast tumors resistant to multiple lines of endocrine therapies. This suggests that *ESR1* fusions may be enriched in tumors from the selective pressure of endocrine treatment. Since the ESR1-e6>YAP1 and ESR1-e6>PCDH11X fusions were identified from a small cohort of late-stage ER+ patients, more RNA-seq data from primary and late-stage, treatment-refractory tumors are clearly required, particularly with longer sequencing reads, which increase fusion gene detection sensitivity to better understand the incidence of *ESR1* fusions in both primary and metastatic breast cancer.

The underlying mechanism of how *ESR1* fusions arise remains unclear. However, as mentioned earlier, DSBs mediated by recruitment of TOP2B to ER transcriptional complexes may contribute to formation of *ESR1* fusion genes, and therefore TOP2B could potentially be an attractive therapeutic target to prevent the formation of *ESR1* fusion events. More studies are required to test this hypothesis. Daunorubicin, an FDA-approved chemotherapeutic drug indicated for treating leukemia, targets TOP2B, however, this agent is very toxic. Developing less toxic agents that target TOP2B may represent a therapeutic strategy to prevent *ESR1* translocation events and deserves further study in the context of ER+ breast cancer.

Therapeutic targeting these aberrant forms of ER have shown promise in pre-clinical experimental models with more studies required to translate such findings to the clinic. Collectively, these studies deepen our understanding of how *ESR1* alterations trigger breast cancer to become lethal metastatic disease and will guide development of therapeutic strategies to treat a subset of patients with tumors that contains these *ESR1* alterations.
